# The Role of the Subcostal Transversus Abdominis Plane Block in Facilitating Operating Room Extubation After Living Donor Liver Transplantation for Hepatocellular Carcinoma: A Propensity Score-Matching Analysis

**DOI:** 10.3390/life15020297

**Published:** 2025-02-14

**Authors:** Jaewon Huh, Min Suk Chae

**Affiliations:** Department of Anesthesiology and Pain Medicine, Seoul St. Mary’s Hospital, College of Medicine, The Catholic University of Korea, Seoul 06591, Republic of Korea

**Keywords:** subcostal TAP block, living donor liver transplantation, early extubation, operating room

## Abstract

**Background:** Effective pain management is essential to early extubation and recovery in living donor liver transplantation (LDLT). The subcostal transversus abdominis plane (TAP) block has emerged as a potential strategy to address postoperative pain while reducing opioid consumption. This study evaluated the effectiveness of the TAP block in facilitating early extubation in the OR and examined its impact on re-intubation rates, postoperative fentanyl requirements, and pain intensity upon ICU admission to determine its role in perioperative pain management. **Methods:** This retrospective cohort study included adult patients who underwent LDLT for hepatocellular carcinoma within the Milan criteria. Propensity score matching was performed to compare outcomes between patients who received the subcostal TAP block and those who did not. The primary outcome was the rate of successful extubation in the operating room (OR). Secondary outcomes included re-intubation rates, postoperative fentanyl requirements, and peak numeric rating scale (NRS) pain scores upon ICU admission. **Results:** The subcostal TAP block was associated with a significantly higher rate of successful OR extubation compared with no TAP block. Multivariable analysis revealed that the TAP block independently increased the likelihood of successful extubation. Patients receiving the TAP block required less fentanyl for pain management and demonstrated lower peak NRS pain scores upon ICU admission. No complications related to the TAP block were observed, underscoring its safety in this high-risk population. **Conclusions:** The subcostal TAP block facilitates early OR extubation by effectively managing postoperative pain and reducing opioid requirements, promoting smoother recovery without increasing adverse events. These findings support its inclusion in multimodal analgesia protocols for optimizing perioperative outcomes in LDLT patients.

## 1. Introduction

Advancements in perioperative care have markedly improved outcomes for liver transplantation (LT), yet challenges persist in achieving early postoperative rehabilitation. Managing pain effectively after surgery is crucial to speeding recovery and enhancing patient outcomes. However, pain control in LT patients is particularly complex due to impaired liver function and the frequent occurrence of renal dysfunction. Postoperative pain is a well-recognized issue in LT patients, with studies reporting moderate to severe pain in the early postoperative period, contributing to delayed recovery, prolonged ventilation, and increased ICU stay [1]. While opioids are often necessary for postoperative pain management, their use can lead to sedation, respiratory depression, nausea, and vomiting, which may delay recovery and increase the risk of complications [2,3]. Given these concerns, regional anesthesia techniques have gained attention as opioid-sparing strategies for postoperative pain control. Among them, the transversus abdominis plane (TAP) block has emerged as an effective option for managing pain after abdominal procedures, reducing opioid consumption and potentially enhancing recovery outcomes [4,5]. The subcostal TAP block, introduced by Hebbard et al., involves the administration of local anesthetics between the rectus abdominis and transversus abdominis muscles or, sometimes, in the posterior rectus sheath. This approach provides targeted analgesia to the T7 to T12 dermatomes [6,7,8]. The subcostal TAP block has been shown to be effective for upper-abdominal surgeries, such as open liver resections, and has demonstrated potential for use in LT [9,10]. A retrospective analysis of orthotopic LT patients revealed that the subcostal TAP block significantly reduced postoperative morphine requirements, highlighting its feasibility and clinical advantages [11]. Similarly, a prospective study by Assefi et al. demonstrated that the block reduced opioid consumption without compromising pain relief and reported no associated complications, reinforcing its safety and effectiveness [12].

Postoperative management for LT has traditionally relied on prolonged mechanical ventilation and sedation in the intensive care unit (ICU) [13]. However, findings from other surgical fields indicate that early tracheal extubation can significantly improve recovery and overall outcomes [14,15]. Additionally, ICU ventilator support constitutes a major contributor to the healthcare costs associated with LT, and successful early extubation has the potential to mitigate this financial burden [16]. Despite strong evidence supporting the safety and feasibility of early extubation after LT, many transplant centers remain reliant on routine postoperative mechanical ventilation in the ICU. This reliance often stems from concerns about the complexity of the procedure and potential risks, including cardiopulmonary complications, reoperation, failed extubation, and challenges in recovering from surgical stress [17]. Studies have identified effective pain control as a critical factor in achieving successful early extubation in the operating room (OR) after LT, emphasizing its feasibility and clinical benefits [2,12,17,18]. Similar observations in cardiac surgery have demonstrated the effectiveness of fascial plane blocks, such as the erector spinae plane block and the pecto-intercostal fascial block, as components of multimodal analgesia protocols. These techniques have been shown to improve patient comfort, shorten extubation times, and promote faster recovery and rehabilitation [19,20,21].

This study aimed to assess the effectiveness of the subcostal TAP block in promoting early extubation in the OR among patients undergoing living donor liver transplantation (LDLT). Additionally, it examined the block’s impact on related clinical outcomes, such as the necessity for re-intubation, postoperative fentanyl requirements, and pain intensity at the time of ICU admission.

## 2. Patient and Methods

### 2.1. Ethical Consideration

This study was designed as a retrospective observational cohort and conducted in alignment with the ethical standards outlined in the Declaration of Helsinki. Approval for the research protocol was obtained from the Institutional Review Board and Ethics Committee (approval number KC21IRSI0576) on 17 September 2021. Given its retrospective nature, the requirement for obtaining informed consent from participants was waived. The results are reported following the recommendations provided by the Strengthening the Reporting of Observational Studies in Epidemiology (STROBE) guidelines.

### 2.2. Study Population

This study included adult patients aged 19 years or older who underwent elective LDLT at our hospital between September 2012 and September 2022. Eligible participants were those diagnosed with hepatocellular carcinoma (HCC) meeting the Milan criteria and deemed suitable for regional anesthesia, defined as having an international normalized ratio (INR) ≤ 2.0 and a platelet count ≥ 50 × 10⁹/L [22,23]. Exclusion criteria encompassed pediatric patients (<19 years), individuals with other underlying liver disease etiologies (e.g., alcohol-induced, autoimmune, biliary pathology, acute hepatitis A, or drug-/toxin-related liver disease), and those with contraindications to regional anesthesia, such as active skin infections at the injection site or known hypersensitivity to local anesthetics. Patients on anticoagulant or antiplatelet therapy (e.g., aspirin and warfarin) within seven days prior to surgery were also excluded. Additional exclusions included cases involving deceased donor liver transplantation, ABO-incompatible LDLT, multi-organ transplants that included the liver, or re-transplantation. Patients with significant preoperative systemic comorbidities, such as uncontrolled cardiac, pulmonary, or renal conditions that contraindicated LDLT, were also omitted. Similarly, those requiring prolonged postoperative sedation (>24 h) or sedation reintroduction within 48 h, which could interfere with recovery assessments, were excluded. Finally, patients with incomplete or missing key data regarding recipient or donor graft characteristics were not included in this study.

During the study period, 677 patients underwent LDLT at our institution. After applying the inclusion and exclusion criteria, 388 patients were excluded, primarily due to non-HCC liver disease (190 patients), contraindications to regional anesthesia (123 patients), prolonged postoperative sedation requirements (70 patients), and incomplete or missing clinical data (5 patients), leaving 289 eligible patients for analysis.

**Figure 1:**
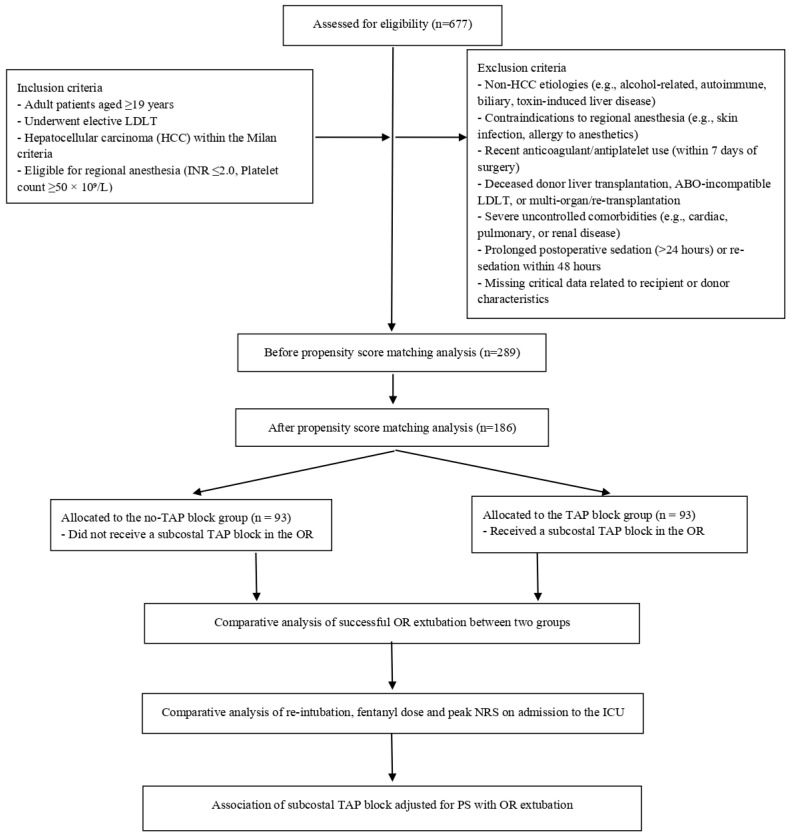
Study flow diagram.

To minimize potential confounding, propensity score (PS) matching was performed, yielding a final cohort of 186 matched patients. These patients were evenly divided into two groups based on the application of the subcostal TAP block, with 93 patients in the TAP block group and 93 in the no-TAP block group (Figure 1).

### 2.3. Surgical and Anesthetic Management

The recipient’s surgical procedure and anesthetic management have been detailed in our prior studies [24,25]. In summary, the recipient surgery involved a J-shaped subcostal incision, followed by implantation of the donor liver. The piggyback technique was employed to optimize venous drainage without the need for complete caval clamping. To ensure proper hepatic venous outflow, reconstruction of the middle hepatic vein was performed as needed. After completing hepatic vessel and bile duct anastomoses, Doppler ultrasonography was used to confirm vascular patency and assess graft perfusion before closing the abdominal incision.

Anesthesia was managed by using a combination of desflurane, rocuronium, and remifentanil to achieve optimal muscle relaxation and maintain hemodynamic stability. Perioperative care was guided by invasive hemodynamic monitoring, including measurements of arterial blood pressure, central venous pressure, and cardiac output. Hemodynamic instability was managed with targeted fluid resuscitation and the careful use of vasopressors to maintain systemic perfusion pressures. Diuretics were administered as needed to address oliguria, fluid overload, or electrolyte imbalances, ensuring stable systemic hemodynamics.

To optimize postoperative analgesia while minimizing opioid use, a multimodal analgesia protocol was implemented. This included a combination of systemic analgesics and a subcostal TAP block, which was performed immediately before awakening at the conclusion of surgery. The subcostal TAP block was integrated into the analgesic strategy to provide targeted pain relief for the J-shaped subcostal incision, facilitating early extubation and reducing opioid requirements.

Postoperative analgesia was initiated at the time of surgical site closure, approximately 60 min before the planned extubation, using a combination of nefopam (1 mg/kg) and fentanyl (1 μg/kg) diluted in 100 mL of normal saline, provided there were no contraindications. Upon ICU admission, peak pain levels were assessed by using the numeric rating scale (NRS). Rescue analgesia with fentanyl (50 μg) was administered as needed, depending on the NRS score. For scores ≥ 5, additional fentanyl was provided every 15 min until the pain score fell below 5. All fentanyl doses were determined by the attending ICU physician, who was not involved in the study, and meticulously recorded by nursing staff.

### 2.4. Subcostal TAP Block

Since September 2012, the subcostal TAP block has been integrated into the perioperative management of LDLT patients as part of a multimodal analgesia strategy to enhance postoperative pain control and reduce opioid consumption. Introduced as an opioid-sparing technique, the TAP block aims to improve recovery outcomes while minimizing opioid-related adverse effects. It was selectively performed in LDLT patients deemed suitable for regional anesthesia, ensuring both safety and procedural efficacy, while those with significant coagulation abnormalities or contraindications to regional anesthesia were excluded.

In the OR, before awakening, a subcostal TAP block was performed by a single anesthesiologist with expertise in regional anesthesia to provide targeted pain relief for the J-shaped subcostal incision used in LDLT. The block was administered under real-time ultrasound guidance to ensure precision and reduce the risk of complications. With the patient in a supine position and the abdominal area aseptically prepared, a high-frequency linear ultrasound probe was placed along the subcostal margin on the surgical side, aligned parallel to the costal arch. The anatomical layers, including the external oblique, internal oblique, and transversus abdominis muscles, were clearly identified. An in-plane technique was used to advance a 21-gauge, 100 mm echogenic block needle under ultrasound guidance in a medial-to-lateral direction, targeting the fascial plane between the internal oblique and transversus abdominis muscles. Hydrodissection with 2–3 mL of normal saline was performed to confirm accurate needle placement within the fascial plane. Following confirmation, 20 mL of 0.25% ropivacaine was incrementally injected to ensure adequate spread and effective analgesic coverage of the incision site. The block was administered unilaterally on the side of the J-shaped incision and completed at the conclusion of surgery. Throughout the procedure, the patient was carefully monitored for signs of local anesthetic systemic toxicity (LAST) and other potential complications to ensure safety.

### 2.5. OR Extubation Criteria

In this study, decisions regarding extubation in the OR after LT were made by experienced anesthesiologists specializing in liver transplant anesthesia [24]. Extubation readiness was assessed based on a standardized set of criteria (Supplementary Table S1). Adequate oxygenation was defined as a peripheral oxygen saturation (SpO_2_) of at least 95% with a fractional inspired oxygen concentration (FiO_2_) of 0.5 or less. Ventilatory adequacy required a tidal volume greater than 5 mL/kg, a spontaneous respiratory rate below 25 breaths per minute, and end-tidal carbon dioxide (ETCO_2_) levels within the normocarbic range of 30–40 mmHg. Hemodynamic stability was also essential, with minimal vasopressor support defined as a norepinephrine infusion rate of less than 0.1 μg/kg/min. Full reversal of neuromuscular blockade was confirmed through indicators such as a sustained head lift or a firm hand grasp. Neurological assessments ensured that patients could respond to simple verbal commands, had spontaneous eye opening, and retained protective reflexes like coughing and gagging. Metabolic stability requirements included a pH above 7.25, normal serum electrolyte levels, and evidence of euvolemia. A core body temperature of at least 35.5 °C was also required. Surgical considerations were reviewed, and extubation was performed only in the absence of significant bleeding or concerns about hepatic vascular patency. Routine consultation with the surgical team was not necessary unless specific complications arose. After extubation, all patients were transferred to the ICU for postoperative monitoring and care.

### 2.6. Outcomes

The primary outcome of this study was the rate of successful extubation in the OR immediately following LT. Successful extubation was defined as meeting all predefined extubation criteria without the need for postoperative mechanical ventilation before transfer to the ICU. Secondary outcomes included the rate of re-intubation within the first postoperative hour, the total fentanyl dose administered for pain management upon ICU admission, and the peak numeric rating scale (NRS) pain score recorded at the time of ICU admission. Re-intubation was noted if mechanical ventilation was required due to inadequate oxygenation, insufficient ventilation, hemodynamic instability, or other clinical concerns. Fentanyl dosage was documented to evaluate postoperative analgesic needs, and the peak NRS score served as an objective measure of immediate postoperative pain severity.

### 2.7. Clinical Variables for PS-Matching Analysis

For the PS-matching analysis, clinical variables were carefully chosen to ensure comparability between the subcostal TAP block group and the no-TAP block group. These variables included preoperative recipient characteristics, intraoperative parameters, and donor graft-related factors to address potential confounders and create balanced groups for analysis. Baseline demographic and clinical characteristics included sex, age, body mass index (BMI), and comorbidities such as hypertension and diabetes mellitus. Liver disease severity and complications were assessed by using the Model for End-Stage Liver Disease (MELD) score, history of varices, and the presence of ascites (≥1 L). Preoperative cardiac function was evaluated through echocardiographic findings, including ejection fraction (%) and diastolic dysfunction (≥grade II). Laboratory parameters considered included white blood cell count, hematocrit, aspartate aminotransferase (AST), alanine aminotransferase (ALT), serum sodium, potassium, albumin, ammonia, platelet count, and INR. Intraoperative factors included the total surgery duration and average vital signs, such as systolic and diastolic blood pressure and heart rate. Transfusion requirements were documented, including the number of packed red blood cell (PRBC) units and fresh frozen plasma (FFP) units administered. Donor graft-related variables included donor sex, age, and BMI, along with graft-specific factors such as the graft–recipient weight ratio (%), graft weight (g), fatty percentage (%), and total ischemic time (minutes). These variables were used in the PS-matching process to minimize baseline discrepancies between the groups, enabling a reliable comparison of perioperative outcomes associated with the subcostal TAP block.

### 2.8. Statistical Analysis

The Shapiro–Wilk test was used to evaluate the normality of continuous variables. Continuous data are reported as medians with interquartile ranges, while categorical data are presented as counts and percentages. To reduce confounding factors between the TAP block and no-TAP block groups, PS matching was applied. Propensity scores were calculated, and one-to-one matching was performed by using a greedy algorithm without replacement to achieve balance between the groups. Perioperative recipient and donor graft variables were analyzed by using the Mann–Whitney U test for continuous variables. For categorical variables, either the χ^2^ test or Fisher’s exact test was employed, depending on the data distribution and sample size. The effect of the subcostal TAP block on successful extubation in the OR was assessed through multivariable logistic regression analysis, adjusted for the PS. Results are presented as odds ratios with 95% confidence intervals, and a *p*-value of <0.05 was considered statistically significant. All statistical analyses were conducted by using SPSS for Windows (version 24.0; IBM Corp., Armonk, NY, USA) and Microsoft Excel.

## 3. Results

### 3.1. Demographic Variables

All patients had HCC within the Milan criteria and underwent elective LDLT. Among the 289 patients, 240 (83.0%) were male, with a mean age of 55.2 ± 7.5 years and a mean BMI of 24.6 ± 3.6 kg/m^2^. Hypertension was observed in 68 patients (23.5%), and diabetes mellitus in 79 patients (27.3%). The mean MELD score was 11.6 ± 6.5 points. A history of varices was present in 49 patients (17.0%), and ascites was identified in 96 patients (33.2%). The mean PLT count was 150.9 ± 37.3 × 10^9^/L, and the mean INR was 1.5 ± 0.5.

### 3.2. PS-Matching Analysis of Pre- and Intraoperative Recipient and Donor/Graft Variables According to Subcostal TAP Block

Before PS matching, several significant differences were observed between the no-TAP and TAP block groups (Table 1). Patients in the TAP block group had a lower median age (54.0 vs. 56.5 years, *p* = 0.013), higher MELD scores (13.6 vs. 6.7, *p* < 0.001), and lower hematocrit levels (30.1% vs. 34.3%, *p* < 0.001). Additionally, the TAP block group had higher ALT levels (median of 32.0 IU/L vs. 29.0 IU/L, *p* = 0.002) and lower albumin levels (median of 3.0 g/dL vs. 3.4 g/dL, *p* < 0.001). Comorbid hypertension was less prevalent in the TAP block group (16.6% vs. 30.6%, *p* = 0.005).

After PS matching, these variables were balanced between the no-TAP and TAP block groups. There were no significant differences in demographic characteristics, MELD scores (median of 7.9 vs. 8.2, *p* = 0.888), or laboratory parameters, such as hematocrit (33.0% vs. 33.5%, *p* = 0.660) and albumin levels (3.3 g/dL vs. 3.2 g/dL, *p* = 0.742). Furthermore, intraoperative variables, including operation time and transfusion requirements, showed no statistically significant differences between the two groups after matching.

### 3.3. OR Extubation and ICU Outcomes

**Figure 2:**
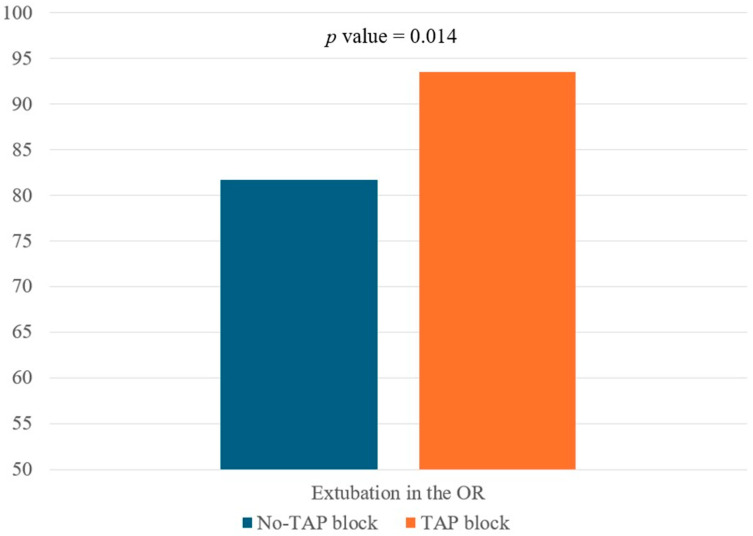
Operating room extubation rates in patients undergoing living donor liver transplantation, stratified by TAP block intervention. Patients in the TAP block group had a significantly higher rate of successful extubation in the operating room compared with the no-TAP block group (93.5% vs. 81.7%; *p* = 0.014). Abbreviations: TAP, transversus abdominis plane; OR, operating room.

In the PS-matched cohort of 186 patients (93 in each group), the rate of extubation in the OR was significantly higher in the TAP block group compared with the no-TAP block group (93.5% vs. 81.7%; *p* = 0.014). This finding, as shown in Table 2 and Figure 2, demonstrates a statistical association between the subcostal TAP block and successful OR extubation.

The rate of re-intubation upon admission to the ICU was lower in the TAP block group (1.1% vs. 3.2%), although this difference was not statistically significant (*p* = 0.621). Re-intubation in the no-TAP block group was primarily attributed to CO_2_ retention caused by shallow tachypnea due to incisional pain, which disrupted deep and smooth breathing. In the TAP block group, a single case of re-intubation occurred, potentially due to incomplete analgesic coverage provided by the block. In all instances, re-intubation was resolved within one hour, with patients successfully weaned from ventilatory support and discharged from the ICU without further complications.

Patients in the TAP block group required significantly lower fentanyl doses upon ICU admission (median of 50 μg vs. 150 μg; *p* < 0.001) and reported lower peak visual analog scale (VAS) pain scores (median of 6 vs. 8; *p* < 0.001), reflecting improved postoperative pain management.

### 3.4. Association Between Subcostal TAP Block Adjusted for PS and OR Extubation

Multivariate analysis adjusted for PS demonstrated a significant association between the subcostal TAP block and successful OR extubation. The TAP block was independently associated with a higher likelihood of successful OR extubation, with an odds ratio of 3.243 (95% CI: 1.217–8.644; *p* = 0.019) (Table 3).

### 3.5. Postoperative Subcostal TAP Block-Related Complications

In this study, no complications related to the subcostal TAP block were observed. Specifically, there were no incidences of local anesthetic systemic toxicity (LAST), which could result from inadvertent intravascular injection or excessive systemic absorption of the anesthetic agent. Similarly, no cases of hematoma formation at the injection site were reported, indicating that the procedure did not cause vascular injury.

Potential risks associated with regional anesthesia, such as infection at the injection site, were also absent in this cohort. Moreover, no mechanical complications, including inadvertent puncture of surrounding organs or structures, were documented. These results suggest that the subcostal TAP block was consistently performed without any observable adverse events, further supporting its safety as a perioperative analgesic technique.

## 4. Discussion

This study demonstrates that the subcostal TAP block is a feasible analgesic option in highly selected LDLT patients and is associated with certain perioperative benefits, including improved pain control and reduced opioid consumption. While these findings suggest a potential role for the TAP block in facilitating early extubation, the observational nature of this study and the inclusion of both preoperative and intraoperative selection criteria introduce the possibility of selection bias. Patients who received the TAP block had lower opioid requirements and improved pain scores upon ICU admission. Additionally, while the TAP block group showed a higher rate of successful extubation in the OR, these findings should be interpreted with caution due to the potential influence of confounding factors. The observed lower re-intubation rate in the TAP block group may reflect improved analgesia but could also be influenced by patient selection variables. Importantly, no TAP block-related complications were reported, supporting its safety and feasibility when performed under real-time ultrasound guidance.

Research in cardiac surgery has emphasized the role of fascial plane blocks in facilitating earlier tracheal extubation and reducing ICU stays. Revollo et al. found that patients who received fascial plane blocks, particularly the erector spinae plane block, had significantly shorter extubation times (reduced by 9.29 h; 95% CI: −11.98 to −6.60; *p* = 0.022) and shorter ICU stays by 1.1 days (95% CI: −1.43 to −0.79; *p* < 0.0001) compared with those managed with intravenous analgesia alone, with no complications reported [21]. Similarly, Wang et al. demonstrated that the pecto-intercostal fascial block (PIFB) reduced extubation times (9.4 ± 4.1 h vs. 12.1 ± 4.6 h; *p* = 0.031) and intraoperative opioid use (153.2 ± 48.3 μg vs. 199.4 ± 51.7 μg; *p* = 0.002). The PIFB group also experienced lower coughing pain scores (1.45 ± 1.43 vs. 3.00 ± 1.71; *p* = 0.021) without significant differences in resting pain scores or adverse events [20]. In our study, the subcostal TAP block demonstrated clear clinical benefits in promoting earlier extubation and improving postoperative outcomes in LDLT patients. The TAP block group had a significantly higher rate of successful extubation in the OR compared with the no-TAP block group (93.5% vs. 81.7%; *p* = 0.014). Multivariable analysis further revealed that the TAP block increased the odds of successful extubation in the OR by three times (odds ratio: 3.243; 95% CI: 1.217–8.644; *p* = 0.019). Beyond extubation, the TAP block significantly reduced fentanyl requirements upon ICU admission (median of 50 μg vs. 150 μg; *p* < 0.001) and lowered peak NRS pain scores (median of 6 vs. 8; *p* < 0.001), underscoring its efficacy in pain management. Importantly, no complications related to the TAP block were observed, demonstrating its safety and feasibility when performed under real-time ultrasound guidance [7,8,26]. The observed association between the TAP block and improved perioperative outcomes suggests that regional analgesia techniques may contribute to optimizing postoperative recovery in LDLT patients, particularly by reducing opioid consumption and facilitating early extubation. These effects could lead to better respiratory function, reduced postoperative sedation, and improved hemodynamic stability, all of which are critical in high-risk surgical populations. Furthermore, the findings underscore the importance of individualized pain management strategies in LDLT recipients. Given the variability in hepatic function and coagulation profiles among transplant patients, a targeted multimodal analgesia approach that includes regional techniques like the TAP block may help balance effective pain relief with safety considerations. Identifying optimal candidates for the TAP block through refined selection criteria may further enhance its utility while minimizing the risk of complications.

The concept of accelerated recovery following surgery, known as Enhanced Recovery After Surgery (ERAS), has gained significant attention in recent years. ERAS emphasizes standardized perioperative strategies aimed at optimizing healthcare resource use, minimizing complications, and enhancing patient outcomes [27]. A key aspect of this approach is the incorporation of multimodal analgesic techniques, including advanced regional anesthesia methods such as the pectoral plane block, serratus anterior plane block, and erector spinae plane (ESP) block. In line with ERAS principles, studies have shown that these interventions can reduce recovery times without increasing the risk of adverse events [28,29,30]. Effective pain management, early tracheal extubation, and the avoidance of prolonged mechanical ventilation are critical goals in perioperative care for LT. Adequate pain control not only improves patient comfort but also helps prevent complications related to impaired ventilation. Early extubation, a cornerstone of ERAS protocols, reduces risks associated with ventilator use and accelerates recovery. Avoiding prolonged mechanical ventilation further enhances outcomes by decreasing morbidity and healthcare costs [3,31]. This study aligns with the principles of ERAS, demonstrating the subcostal TAP block as a valuable component of a multimodal strategy to improve patient care in complex surgical procedures like LDLT. By providing effective pain relief, the TAP block minimizes opioid use, reducing associated risks and facilitating early extubation—a critical step toward rapid recovery. Specifically, the subcostal TAP block proved effective in managing pain from the J-shaped incision in LDLT, alleviating parietal pain-induced respiratory compromise. This enabled sufficient tidal volume without tachypnea, ensuring stable respiratory function and reducing the likelihood of prolonged mechanical ventilation. Further research should investigate the long-term benefits of the TAP block and refine protocols to enhance its application in liver transplantation.

The subcostal TAP block employed in this study is a regional anesthesia technique that targets the fascial plane between the transversus abdominis and internal oblique muscles. By delivering local anesthetic to this plane, the block interrupts nerve conduction in the anterior branches of the lower thoracic intercostal nerves (T7–T12) and, in some cases, the iliohypogastric and ilioinguinal nerves [7,8]. This technique is particularly effective for managing pain in dermatomes associated with upper-abdominal incisions, making it highly suitable for LT procedures involving J-shaped subcostal incisions. The block’s primary effectiveness in LT lies in its ability to provide localized analgesia for parietal pain, which arises from the abdominal wall incision and prolonged retraction needed for liver exposure. Parietal pain significantly contributes to postoperative discomfort and respiratory challenges, such as limited diaphragmatic movement, shallow breathing, or tachypnea. By alleviating this pain, the subcostal TAP block helps maintain adequate tidal volume and stable respiratory function, reducing the need for extended ventilatory support and enabling earlier extubation [3,16,17,24,32]. For LT patients, who often have impaired liver function and coagulopathy, the TAP block offers a key safety advantage. Unlike neuraxial techniques such as epidural or paravertebral blocks, which carry a heightened risk of hematoma in anticoagulated individuals, the TAP block provides effective analgesia without breaching intrathecal or epidural spaces. Administering the block under real-time ultrasound guidance further minimizes the risk of vascular or visceral injury, ensuring precision and safety [11,12]. Additionally, the opioid-sparing benefits of the TAP block make it especially valuable in this patient population. Reduced opioid use is advantageous as excessive opioids can worsen hepatic encephalopathy, delay gastrointestinal recovery, and heighten the risk of respiratory depression [33,34]. These combined physiological and safety benefits establish the subcostal TAP block as a crucial element of the multimodal analgesic approach in LT, significantly improving postoperative outcomes while maintaining a robust safety profile.

This study has several limitations that should be acknowledged. First, its retrospective design introduces the potential for selection bias, as the administration of the TAP block was determined by both preoperative and intraoperative factors, which may have influenced the observed outcomes. Although propensity score matching was used to minimize confounding, residual biases cannot be entirely ruled out. Second, as a single-center study, the findings may not be fully generalizable to other institutions with different surgical approaches, perioperative protocols, or patient populations. Third, the evaluation of pain control and opioid consumption was limited to the immediate postoperative period, without assessing long-term outcomes such as chronic pain, opioid dependence, or overall quality of life, which could provide a more comprehensive understanding of the TAP block’s impact. Fourth, while the TAP block was performed under ultrasound guidance, differences in operator experience and technique may have influenced its effectiveness and safety, though this aspect was not specifically analyzed. Lastly, this study focused exclusively on LDLT recipients with HCC within transplant eligibility criteria who were considered suitable candidates for regional anesthesia based on coagulation and platelet function assessments. These selection criteria may limit the generalizability of the findings, as the results may not directly apply to LDLT recipients with other liver diseases, patients undergoing deceased donor liver transplantation, or individuals with different perioperative risk profiles. Future research should aim to address these limitations through prospective, multicenter studies with larger cohorts. Additionally, randomized controlled trials with extended follow-up could further clarify the long-term benefits of the TAP block and help refine patient selection criteria for its use in liver transplantation.

## 5. Conclusions

This study demonstrates that the subcostal TAP block is a feasible analgesic technique in highly selected LDLT recipients and is associated with certain perioperative benefits, including improved pain control and reduced opioid requirements. While these findings suggest a potential role for the TAP block in facilitating early extubation and enhancing perioperative recovery, several limitations must be considered. The retrospective study design and selection criteria for TAP block administration introduce the possibility of selection bias, and despite propensity score matching, residual confounding factors cannot be entirely ruled out. Additionally, as a single-center study, the findings may not be fully generalizable to other institutions with different perioperative practices and patient populations. This study also focused on the immediate postoperative period, and long-term analgesic effects, functional recovery, and overall quality of life outcomes were not assessed. Given these limitations, our results should be interpreted with caution, and larger prospective, multicenter studies are needed to confirm its efficacy, refine patient selection criteria, and optimize clinical implementation. Nevertheless, the safety and feasibility of the TAP block, particularly when performed under ultrasound guidance, support its consideration as part of multimodal analgesia protocols in carefully selected LDLT patients. Future research should focus on optimizing dosing strategies, identifying patient subgroups that may derive the greatest benefit, and evaluating its potential role in broader liver transplant populations, including deceased donor liver transplantation and emergency surgical settings.

## Figures and Tables

**Table 1:** Perioperative demographic variables in the no-TAP block and TAP block groups before and after propensity score matching.

	Before Propensity Score Matching				After Propensity Score Matching			
Group	No-TAP Block	TAP Block	*p*-Value	SD	No-TAP Block	TAP Block	*p*-Value	**SD**
**n**	**144**	**145**			**93**	**93**		
***Preoperative recipient variables***								
Sex (male)	122 (84.7%)	118 (81.4%)	0.449	0.086	80 (86.0%)	77 (82.8%)	0.544	0.083
Age (years)	56.5 (52.0–61.0)	54.0 (50.0–59.0)	0.013	−0.262	56.0 (52.0–60.0)	55.0 (51.0–60.0)	0.457	−0.091
BMI (kg/m^2^)	24.2 (22.0–26.4)	24.4 (22.9–26.7)	0.195	0.146	24.1 (22.0–26.7)	24.2 (22.5–26.1)	0.727	0.044
Comorbidity								
Hypertension	44 (30.6%)	24 (16.6%)	0.005	−0.376	24 (25.8%)	19 (20.4%)	0.385	−0.144
Diabetes mellitus	38 (26.4%)	41 (28.3%)	0.719	0.042	24 (25.8%)	26 (28.0%)	0.741	0.048
MELD score (points)	6.7 (6.0–10.9)	13.6 (6.0–21.7)	<0.001	0.754	8.2 (6.0–11.9)	7.9 (6.0–15.0)	0.888	0.094
Complications								
Varix	16 (11.1%)	33 (22.8%)	0.008	0.277	12 (12.9%)	13 (14.0%)	0.83	0.026
Ascites (≥1 L)	42 (29.2%)	54 (37.2%)	0.145	0.166	31 (33.3%)	28 (30.1%)	0.636	−0.066
Echocardiography								
Ejection fraction (%)	64.0 (62.0–66.0)	65.0 (62.3–67.4)	0.096	0.149	64.0 (63.0–66.3)	65.0 (62.7–67.7)	0.249	0.123
Diastolic dysfunction (≥grade II)	22 (15.3%)	28 (19.3%)	0.365	0.102	16 (17.2%)	17 (18.3%)	0.848	0.027
Laboratory values								
White blood cell count (×10^9^/L)	4.1 (2.8–5.2)	3.4 (2.6–5.4)	0.251	0.105	4.1 (2.5–5.2)	3.4 (2.5–4.9)	0.404	0.093
Hematocrit (%)	34.3 (28.7–39.2)	30.1 (25.8–36.1)	<0.001	−0.401	33.5 (27.5–37.7)	33.0 (27.1–37.9)	0.66	−0.040
Aspartate aminotransferase (IU/L)	34.5 (26.3–50.8)	50.0 (33.5–84.5)	<0.001	0.358	34.0 (26.0–51.0)	39.0 (29.0–61.5)	0.076	0.064
Alanine aminotransferase (IU/L)	29.0 (20.0–42.0)	32.0 (24.0–67.0)	0.002	0.323	28.0 (18.0–41.5)	31.0 (21.5–44.5)	0.126	0.048
Sodium (mEq/L)	141.0 (138.0–142.0)	139.0 (136.0–141.0)	<0.001	−0.305	141.0 (137.0–142.0)	140.0 (137.0–142.0)	0.549	−0.027
Potassium (mEq/L)	4.0 (3.7–4.3)	4.0 (3.7–4.3)	0.601	−0.031	4.0 (3.8–4.3)	4.0 (3.8–4.3)	0.575	−0.040
Albumin (g/dL)	3.4 (2.8–3.9)	3.0 (2.6–3.6)	<0.001	−0.461	3.2 (2.8–3.8)	3.3 (2.7–3.7)	0.742	−0.056
Ammonia (μg/dL)	89.0 (62.5–134.8)	106.0 (71.0–166.0)	0.018	0.252	96.0 (68.5–143.0)	91.0 (65.0–133.0)	0.417	−0.060
Platelet count (×10^9^/L)	151.0 (130.3–169.0)	147.0 (131.0–162.0)	0.28	−0.196	145.0 (126.5–161.0)	147.0 (128.0–162.0)	0.662	0.052
International normalized ratio	1.3 (1.1–1.4)	1.7 (1.2–2.2)	<0.001	0.832	1.3 (1.1–1.4)	1.2 (1.2–1.6)	0.24	0.098
***Intraoperative recipient variables***								
Operation time (min)	482.5 (435.0–540.0)	500.0 (460.0–595.0)	0.023	0.269	505.0 (442.5–545.0)	490.0 (457.5–572.5)	0.82	0.004
Average of vital signs								
Systolic blood pressure (mmHg)	106.3 (99.3–116.9)	108.3 (97.6–118.4)	0.91	0.016	104.0 (97.5–114.4)	107.5 (97.5–118.4)	0.257	0.153
Diastolic blood pressure (mmHg)	57.7 (53.1–63.7)	58.8 (52.8–64.9)	0.649	0.062	57.0 (52.0–63.8)	58.5 (52.0–64.4)	0.636	0.094
Heart rate (beats/min)	88.3 (80.1–97.9)	87.5 (78.5–100.6)	0.738	−0.022	88.5 (80.3–103.7)	87.3 (77.0–98.0)	0.252	−0.161
Blood transfusion								
Packed red blood cells (units)	5.0 (5.0–5.0)	5.0 (5.0–5.0)	0.746	0.049	5.0 (5.0–5.0)	5.0 (5.0–5.0)	0.444	0.088
Fresh frozen plasma (units)	5.0 (5.0–5.0)	5.0 (5.0–5.0)	0.099	0.284	5.0 (5.0–5.0)	5.0 (5.0–5.0)	0.798	0.094
***Donor graft variables***								
Sex (male)	87 (60.4%)	91 (62.8%)	0.682	−0.048	53 (57.0%)	57 (61.3%)	0.551	−0.089
Age (years)	35.2 (28.0–46.0)	31.0 (24.0–46.5)	0.023	−0.221	34.0 (27.0–41.5)	31.0 (24.5–48.0)	0.418	−0.052
BMI (kg/m^2^)	20.2 (18.2–21.4)	20.2 (18.0–22.0)	0.771	0.008	20.2 (18.2–21.3)	20.2 (17.9–22.5)	0.729	0.062
Graft–recipient weight ratio (%)	1.2 (1.0–1.5)	1.1 (1.0–1.4)	0.075	−0.248	1.2 (1.0–1.4)	1.1 (1.0–1.4)	0.776	−0.061
Graft weight (g)	826.0 (692.5–926.0)	792.0 (669.0–900.0)	0.211	−0.119	800.0 (672.0–896.6)	802.0 (680.0–901.0)	0.977	−0.024
Fatty percentage (%)	4.9 (1.0–5.0)	5.0 (1.0–5.0)	0.156	0.176	4.9 (0.5–5.0)	5.0 (0.5–5.0)	0.485	0.153
Total ischemic time (min)	80.0 (63.5–113.8)	94.0 (67.0–119.5)	0.119	0.022	85.0 (65.5–117.0)	94.0 (66.0–125.5)	0.327	0.053

Abbreviations: TAP, transversus abdominis plane; BMI, body mass index; SD, standard deviation; MELD, model for end-stage liver disease. Values are expressed as numbers (percentages) and medians (interquartile ranges).

**Table 2:** Operating room extubation outcomes in propensity score-matched patients comparing no-TAP and TAP block groups.

Group	No-TAP Block	TAP Block	*p*-Value
n	93	93	
Extubation in the OR (%)	76 (81.7%)	87 (93.5%)	0.014
Re-intubation on admission to the ICU (%)	3 (3.2%)	1 (1.1%)	0.621
Fentanyl dose on admission to the ICU (μg)	150 (150–200)	50 (50–100)	<0.001
Peak NRS on admission to the ICU	8 (8–9)	6 (6–7)	<0.001

Abbreviations: TAP, transversus abdominis plane; OR, operating room; ICU, intensive care unit; NRS, numeric rating scale. Values are expressed as numbers (percentages) and medians (interquartile ranges).

**Table 3:** Association of subcostal TAP block with facilitating operating room extubation in propensity score-matched patients.

	*β*	Odds Ratio	95% CI	*p*-Value
***Subcostal TAP Block Adjusted for PS***				
Successful extubation in the operating room	1.177	3.243	1.217–8.644	0.019

Abbreviations: TAP, transversus abdominis plane; PS, propensity score; CI, confidence interval.

## Data Availability

Data are contained within the article.

## Supplementary Materials

The following supporting information can be downloaded at: https://www.mdpi.com/article/10.3390/life15020297/s1, Supplementary Table S1: Criteria for extubation in the operating room following living donor liver transplantation.
