# Supplementary material for: The Role of the Subcostal Transversus Abdominis Plane Block in Facilitating Operating Room Extubation After Living Donor Liver Transplantation for Hepatocellular Carcinoma: A Propensity Score-Matching Analysis
Source: Life (Basel). 2025 Feb 14;15(2):297. doi: 10.3390/life15020297 (PMC11857526; doi:10.3390/life15020297)
Supplement: Supplementary file 1 [file life-15-00297-s001.zip › life-3433067-supplementary.pdf]

Supplementary Table S1: Criteria for extubation in the operating room following living donor liver transplantation

Category	Criteria
**Oxygenation**	$SpO_2 \geq 95\%$ with $FiO_2 \leq 0.5$
**Ventilation**	Tidal volume > 5 mL/kg, respiratory rate < 25 breaths/min, $ETCO_2$ within 30–40 mmHg
**Hemodynamic Stability**	Minimal vasopressor support (norepinephrine < 0.1 μg/kg/min)
**Neuromuscular Function**	Sustained head lift or firm hand grasp
**Neurological Status**	Ability to follow verbal commands, spontaneous eye opening, intact protective reflexes
**Metabolic Stability**	pH > 7.25, normal serum electrolytes, evidence of euvolemia
**Temperature**	Core body temperature ≥ 35.5°C
**Surgical Considerations**	No ongoing bleeding or hepatic vascular patency concerns
**Surgeon Consultation**	Not routinely required unless specific surgical issues arise
**Post-Extubation Care**	Immediate transfer to the ICU for postoperative monitoring and care
